# Assessment of Stakeholder Perceptions and Attitudes Toward Health Data Governance Principles in Botswana: Web-Based Survey

**DOI:** 10.2196/41408

**Published:** 2023-03-13

**Authors:** Kagiso Ndlovu, Kabelo Leonard Mauco, Star Chibemba, Steven Wanyee, Tom Oluoch

**Affiliations:** 1 Department of Computer Science University of Botswana Gaborone Botswana; 2 Department of Health Information Management Botho University Gaborone Botswana; 3 Medical Records Department Sidilega Private Hospital Gaborone Botswana; 4 Department of Computing and Informatics University of Nairobi Nairobi Kenya; 5 School of Public Health University of Ghana Accra Ghana

**Keywords:** health data, governance, Botswana, digital health, decision-making, health care stakeholders, perceptions, health policy, data governance, data policy, implementation

## Abstract

**Background:**

The use of information and communication technologies for health—eHealth—is described as having potential to improve the quality of health care service delivery. Consequently, there is an increased global trend toward adoption of eHealth interventions by health care systems worldwide. Despite the proliferation of eHealth solutions, many health care institutions especially in transitioning countries are struggling to attain effective data governance approaches. The Ministry of Health in Botswana is an exemplar institution continually seeking better approaches to strengthen health data governance (HDG) approaches following the adoption of eHealth solutions. Recognizing the need for a global HDG framework, the Transform Health coalition conceptualized HDG principles that are structured around 3 interconnected objectives: protecting people, promoting the value of health, and prioritizing equity.

**Objective:**

The aim of the study is to solicit and evaluate perceptions and attitudes of health sector workers in Botswana toward the HDG principles by Transform Health and derive any future guidance.

**Methods:**

Purposive sampling was used to select participants. A total of 23 participants from various health care organizations in Botswana completed a web-based survey and 10 participated in a follow-up remote round-table discussion. The aim of the round-table discussion was to gain further insight into participants’ responses from the web-based survey. Participants were from the following health care cadres: nurses, doctors, information technology professionals, and health informaticians. Both validity and reliability testing were performed for the survey tool before sharing it with study participants. An analysis of participants’ close-ended responses from the survey was performed using descriptive statistics. Thematic analysis of open-ended responses from the questionnaire and the round-table discussion was achieved using the Delve software and the widely accepted principles of thematic analysis.

**Results:**

Although some participants highlighted having measures in place similar to the HDG principles, there were some who either did not know or disagreed that their organizations already had in place mechanisms similar to the proposed HDG principles. Participants further expressed relevance and importance of the HDG principles in the context of Botswana. However, some modifications to the principles were also suggested.

**Conclusions:**

This study highlights the necessity of data governance in health care particularly toward meeting the requirements for Universal Health Coverage. The existence of other health data governance frameworks calls for a critical analysis to assess the most appropriate and applicable framework in the context of Botswana and similar transitioning countries. An organization-centered approach may be most appropriate, as well as strengthening of existing organizations’ HDG practices with the Transform Health principles.

## Introduction

Efforts to improve the quality of health care service delivery using information and communication technologies (ICT) have resulted in health systems faced with challenges such as data overload, security, and privacy concerns [1]. Unforeseen access to health data by unauthorized parties also raises concerns with confidentiality and inappropriate data use, including result commercialization and undisclosed surveillance [2]. In addition, the growing data volumes from diverse digital health sources may cause data inconsistencies that need to be identified and addressed before decisions in a health care organization are made based on incorrect data [3]. Notwithstanding these challenges, there is a growing appreciation of “data as an asset” by health care institutions as they face increasing pressure for reporting a “single version of the truth” [4]. The COVID-19 pandemic has further highlighted the importance of data quality for guiding decision-making and practicing evidence-based health care [5].

Data quality—the degree to which a given data set meets a user’s requirements [6]—is essential for use in patient care as well as for monitoring the performance of health care services and the health human resource [7]. Consequently, the need for strengthening health data governance (HDG) has arisen, as noted in pronouncements such as the Sustainable Development Goals [8] and Universal Health Coverage (UHC) [9]. The Data Governance Institute (DGI) defines data governance as “a system of decision rights and accountabilities for information-related processes, executed according to agreed-upon models which describe who can take what actions with what information, and when, under what circumstances, using what methods” [10]. In health care, data governance includes monitoring and enforcing security of critical health information, policies, and procedures to guide, manage, protect, and govern electronic data under the control of a health care facility [11].

The benefits of strong data governance initiatives are many and diverse, and so are the challenges. Some of the documented barriers in establishing data governance best practices in health care include lack of executive support; inadequate resources; little trust in the data; lack of a strategy for educating, training, and supporting users on data governance practices; inconsistent data protections; resistance to change; digital data being perceived as a technology asset and not a corporate asset; complex nature of health care data (mostly unstructured); and noninteroperable data systems [12]. Inadequate data governance practices in any organization consequently results in challenges such as loss of accountability, poor data quality, fragmented ownership with little authority, and nonexistent standards, policies, and procedures. This scenario could result in patients’ data being exposed to exploitation and potentially resulting in bad decisions being made, financial wastage, and opportunity loss [13].

The Ministry of Health (MOH) in Botswana is mandated with the overall oversight and delivery of health care services. Data at the point of generation are captured through hybrid systems involving a combination of paper-based and electronic systems. Botswana’s health sector still uses multiple data collection and reporting tools. Public and private health facilities in Botswana use separate health information management systems. Further, there are no established feedback mechanisms for ensuring that the data flow process is seamless among all the levels of the health system. There also exist challenges of health human resource capacity on data analytics, hindering the effective use of information for decision-making [14].

In Botswana, the continued proliferation of vertical health information systems (before and after the pandemic) has led to duplication of efforts in a scarcely resourced setting [15]. The Botswana National eHealth Strategy further highlights this by stating that “there is duplication of efforts (EMR and DHIS2 data), data coming from the same source and some of the software are not in real time (Subsection 2.2.2)” [16]. As a result, the meaningful use of health data becomes a tedious and complicated task.

As such, a comprehensive HDG framework is essential to address the aforementioned challenges as faced by many health systems worldwide. Upon this realization, Transform Health (a global coalition of organizations based in Switzerland and dedicated to achieving health for all in today’s digital era) led the development of globally unifying, human rights–based HDG principles [17]. Transform Health was set up to collectively respond to digital health challenges by bringing together local, regional, and global stakeholders from multiple sectors dedicated to achieving UHC in the digital age. It campaigns for and collaborates with individuals—particularly women and young people—and communities who would benefit most from the digital transformation of health systems, as well as the governments, organizations, and institutions that recognize and support the fundamental role of digital technologies and data for improved health. The Transform Health coalition coordinated the development of the HDG principles under the leadership of its Policy Circle, whose members are from various organizations such as Young Experts: Tech 4 Health, Central American Health Informatics Network (RECAINSA), Palladium Group/Health Data Collaborative Digital and Data Governance Working Group, Foundation Botnar, IT for Change, FIND, I-DAIR, Philips/Digital Connected Care Coalition, Jhpiego, and Asia eHealth Information Network (AeHIN) [18], all of which collectively developed the HDG principles. Protection of individuals is often embodied in general data protection laws. However, this is at the core of the HDG principles by Transform Health, which highlights that HDG must include special measures of protection against various kinds of individual and collective harms, including data-driven exploitation, harassment, discrimination, surveillance capitalism, and neocolonialism [18]. This dimension on data governance complements other published health care data governance principles [19,20].

The HDG principles by Transform Health are structured around three interconnected objectives: (1) protecting people, (2) promoting the value of health, and (3) prioritizing equity [18]. The principles are a result of an inclusive and consultative process including 8 workshops, a public consultation, and contributions from over 200 experts globally, as well as relevant sectors and stakeholders [18].

As part of an agenda toward establishing awareness as well as advocacy for the principles in Botswana, Transform Health engaged the authors to solicit and evaluate key health sector stakeholders’ perceptions and attitudes toward the aforementioned HDG principles in Botswana and derive any future guidance.

## Methods

### Overview

In this study, purposive sampling was used to select 30 participants (5 nurses, 5 doctors, 5 IT officers or technicians, 5 IT managers, 5 system analysts, and 5 health informaticians). The inclusion criteria were as follows: being part of the health care system in Botswana and their availability and consent to participate in a web-based survey developed by the authors. Only 23 participants responded to the survey, which had 8 close-ended questions (5 multiple choice and 3 Likert scale) and 2 open-ended questions. A 3-point Likert scale was used for the 3 closed-ended questions related to ranking the HDG principles according to priority (ordinal scale: 1=highest priority, 2=moderate priority, and 3=lowest priority).

In order to ensure that the survey items addressed the objectives of the study and that the question items were not ambiguous, the electronic survey was first reviewed among 5 health sector representatives (2 nurses, 2 medical librarians, and 1 health information technology manager) who were not part of the final study participants. The survey was pretested by 2 health information technology officers before being enhanced through improved branching logic. Feedback from the pilot test was considered and incorporated into the final survey distributed to study participants from April 15, 2022, to May 20, 2022. An analysis of participants’ close-ended responses from the survey was performed using descriptive statistics in Microsoft Excel (version 2013; Microsoft Inc) to determine participants’ perceptions regarding the HDG principles.

A follow-up remote round-table discussion was conducted with some of the survey participants who expressed willingness to elaborate on their survey responses. The intent of the round-table discussions was to gain further insight into participants’ survey responses. This was done using Zoom as it was the common virtual platform that all participants had access to. The discussion was led by a moderator and loosely structured to gain an in-depth view and opinion of participants regarding the principles. Thematic analysis of open-ended responses from the questionnaire and the round-table discussion was performed. This was achieved via the Delve software and using the widely accepted principles of thematic analysis [21]. Example quotes from participants were mapped to the identified themes.

### Ethics Approval

The study was approved by the ethics committee of the University of Botswana (reference UBR/RES/IRB/BIO/325). Initial contact with potential participants was done telephonically to explain to them the objective of the exercise, as well as consent to participate in the study. The consent forms clearly explained the purpose of the study and provided assurance that data would be kept safe and deidentified. Participants were informed of their right to refuse to participate or withdraw from the study at any time. Those who consented to participate were sent an electronic link of the questionnaire (developed by authors) for them to complete as well as web address (URL) to the Transform Health HDG principles. The survey was hosted on the Research Electronic Data Capture (REDCap) system. REDCap is a secure (Health Insurance Portability and Accountability Act and General Data Protection Regulation compliant) system for supporting electronic data capture for research and operational support projects [22]. No compensation was provided, and participants included those beyond known to the authors.

## Results

Study participants were representative of various health care stakeholders in Botswana (Table 1).

Survey participants shared their views on whether (1) the HDG principles are important in their industry, (2) their organizations could benefit from implementing the HDG principles, (3) their organizations already have in place mechanisms similar to the HDG principles, and (4) the HDG principles in their current form are adequate to address issues related to HDG in the context of Botswana.

**Figure 1:**
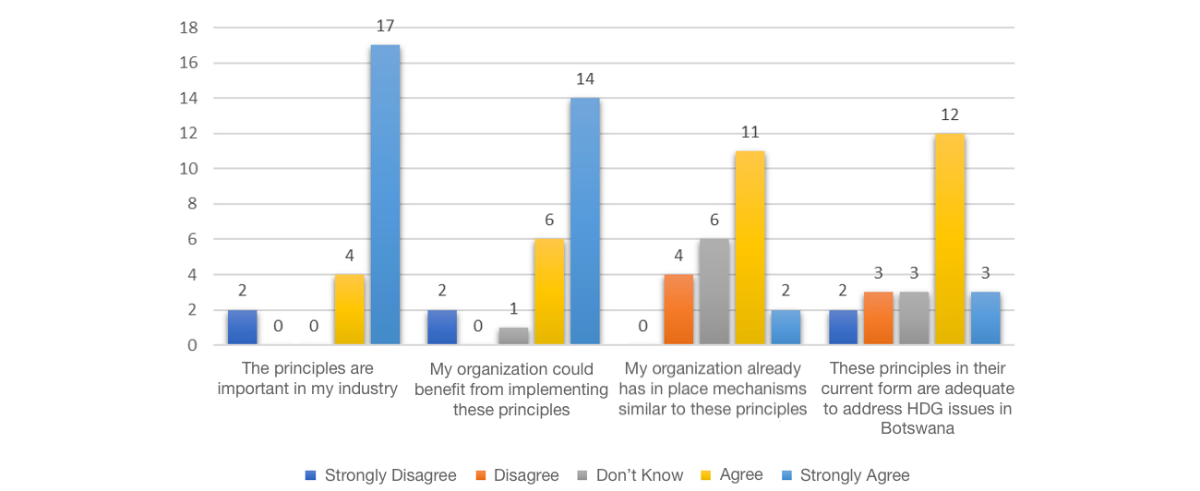
Participants’ perceptions of the importance, benefits, and adequacy of health data governance (HDG) principles in their industry.

Majority of participants (17/23, 74%) strongly agreed that the HDG principles are important in their industries and that their organizations could benefit from their implementation. Although the majority of participants agreed that their organizations already had in place mechanisms similar to the HDG principles, it is worth noting that 17% (4/23) of participants disagreed that their organizations already had in place mechanisms similar to the HDG principles. Lastly, more than half (12/23, 52%) of participants also agreed that the HDG principles in their current form are adequate to address issues related to HDG in the context of Botswana (Figure 1).

**Figure 2:**
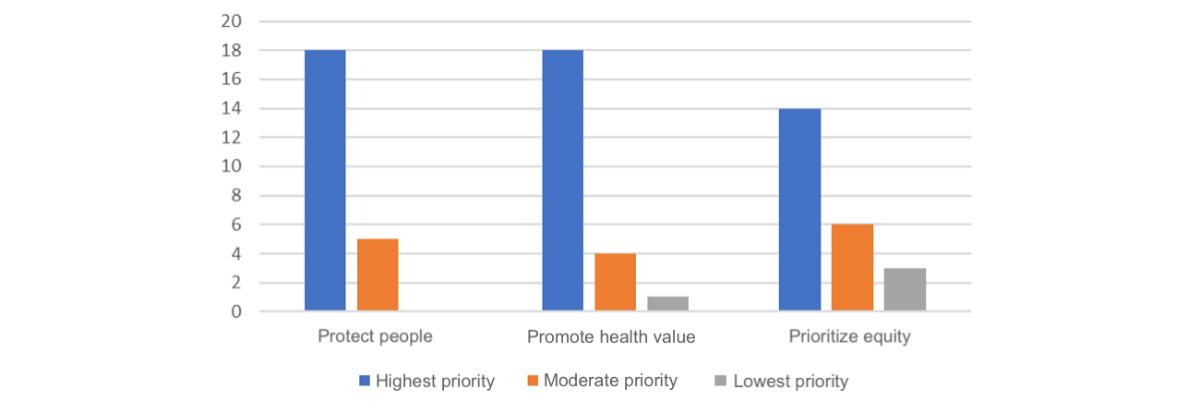
Participants’ ranking of the health data governance principles (lowest to highest priority).

Participants also responded to a question that asked them to rank the HDG principles in order of importance (Figure 2), and none ranked the principle of “protecting people” as being of lowest priority.

A thematic analysis of open-ended responses from the web-based survey and participants’ responses from the round-table discussion regarding the HDG principles are presented in Textboxes 1 and 2. Textbox 1 is a presentation of participants’ opinion on modifications required on the existing HDG principles so that they could better align with the context of Botswana. Textbox 2 is a thematic presentation of participants’ opinion on factors to be considered by an organization willing to implement the HDG principles.

Participants’ proposed revisions to the HDG principles included an elaboration and a suggestion relating to remodeling of the HDG principles (Textbox 1). Participants’ thoughts on factors necessary for successful implementation of the HDG principles by a willing organization resulted in the emergence of 4 themes: needs assessment, data protection, health workforce capacity development, and interoperability (Textbox 2).

**Table 1:** Representation of stakeholders who participated in the health data governance principles survey (N=23).

Stakeholders	Values, n (%)
Ministry of Health	8 (35)
Public health care service provider	1 (4)
Private health care service provider	4 (18)
Tertiary institution	4 (17)
Intergovernmental organization	2 (9)
Other	4 (17)

**Participants’ suggested revisions to the health data governance principles.**

**Elaboration**

- “Detailed clarification of what constitutes health data is required”

**Remodeling**

- “Emphasis on aspects relating to health data accountability and ownership needs to be reflected by the principles”

**Survey participants’ suggestions regarding implementation of health data governance (HDG) principles.**

**Needs assessment (organizational needs before the implementation of the HDG principles)**

- “To start, an evaluation of a given environment to determine the scale at which the principles are needed looking at the current governance values and its impact to this day”
- “Need analysis would be relevant”
- “Private health providers must be engaged”
- “The health system in Botswana is open to innovation. Perhaps a process for new innovations to go through would be useful, in order to be ratified by the ministry of health. This would fast track innovation while ensuring compliance”
- “HDG principles should be treated as equally important”
- “A deliberate decision to transition from paper data to paperless data capture across the country”

**Data protection (organizational data protection considerations before the implementation of the HDG principles)**

- “Health data must be kept within the country and data sharing with other facilities for quick diagnosis and fast treatment of the patient”
- “Data protection is only observed within the private sector and there is no implementation of the data protection act”
- “Protecting people could be strengthened, in terms of confidentiality and protecting info on individual patients, using Omang number (national identity number for citizens) for everything may pose just that risk of one being able to access all confidential info, including health data”

**Health workforce capacity development (human resource capacity development requirements before the implementation of the HDG principles)**

- “Stakeholder awareness campaign workshops are essential”
- “Through workshops, and inclusion of these principles in internal assessments of members”
- “Best would be to educate on international standards, develop procedures and generic templates, legal education around health data and data governance”
- “Capacity needed for Sector leadership on optimal implementation - need for dissemination to implementers”
- “Data managers to be research-centric. This will improve data capture, management and utilisation”

**Interoperability (health data sharing considerations before the implementation of the HDG principles)**

- “There should also be a system that is able to collect health data from the private sector if it is not in place already. I am not aware that there is one. As far as I know clinics keep their own data”
- “Although the ministry of health promotes data sharing through the Health Data Collaborative initiative, there is minimal guidance on how to do that”
- “Enforcing data sharing, intellectual property and data sharing agreements”
- “Implementing health data sharing to avoid duplication”

## Discussion

### Principal Findings

Overall, participants agreed that the HDG principles are important in their industries and that health care institutions could benefit from their implementation. Although some participants highlighted having measures in place similar to the HDG principles, there were some who either did not know or disagreed that their organizations already had in place mechanisms similar to the proposed HDG principles. Participants further agreed that HDG principles in their current form are adequate to address issues related to HDG in the context of Botswana. Each principle was considered to be of high priority by the majority of participants, and none of the participants perceived the principle of “Protecting People” as being of lowest priority. The need to provide a clear definition of what constitutes health data as well as an emphasis on issues relating to health data accountability emerged from the round-table discussions. Participants’ responses on considerations for successful implementation of the HDG principles by a willing organization in Botswana were categorized into 4 themes (needs assessment, data protection, health workforce capacity development, and interoperability).

One participant expressed that “landscape analysis will help to determine the scale at which the principles are needed looking at the current governance values and its impact to this day.” Consequently, the needs assessment process will guide decision-making, justify decisions before they are made, result in flexible processes, and offer solutions to complex problem scenarios [23].

According to the DGI [10], four major factors that may influence an organization’s need to adopt formal data governance include (1) an organization’s expansion such that traditional management is not able to address data-related cross-functional activities; (2) complex data systems making it tedious for traditional management methods to address data-related cross-functional activities; (3) the need for organizational units to interoperate and share data; and (4) regulation, compliance, or contractual requirements for formal data governance. The World Health Organization (WHO) further recognizes the ongoing accelerated trends toward digitization in health, persistent data gaps, and fragmented approaches to governing health data in different contexts, and calls for a global consensus on HDG [24]. The above-cited reasons by both DGI and WHO could justify one of the findings in the current study whereby the majority of participants “strongly agreed” that the HDG principles are important in their industries and that their organizations could benefit from implementing these principles. Furthermore, regulatory obligations such as complying with the Botswana Data Protection Act (DPA) of 2018 [25] and the guiding principles outlined within the Botswana National eHealth Strategy (2020-2024) [16] may have contributed to participants appreciating the importance and the benefit of the HDG principles in their organizations. Compliance to the DPA can be strengthened by making sure that it informs all health data management practices in health care organizations.

It is worth noting that in most organizations, data-related roles exist such as operational, tactical, strategic, and support roles [26]. Consequently, most organizations may be already governing data, but in an informal manner [26]. Therefore, depending on how well individuals are informed with regard to issues of data governance, one may or may not be aware of their organizations’ data governance status or their expected roles and responsibilities regarding data governance in their organizations. This may explain participants’ varying views regarding their organizations’ HDG status. Therefore, health workforce capacity development is essential as it would make implementation of the principles smooth because employees would understand its purpose and hence become less resistant to change. Moreover, continuous training helps organizations to evolve as new challenges and complex scenarios will emerge over time requiring different resolution approaches [27,28]. The train-the-trainer model is documented as an example of a sustainable training model [27]. Moreover, training that is tailor-made to a country’s experiences could inform sustainable approaches to health human resource capacity development. For example, a recent study conducted in Tanzania reported that the MOH successfully coordinated a hands-on training that used a structured methodology and standardized training materials for different groups of users, with participants given access to materials, facilitator-led demonstrations, presentations, group assignments, pretests, and posttests to enhance trainees’ understanding and assimilation of issues [28].

Globally, the increasing number of heterogeneous noninteroperable digital health systems have resulted in data silos across health sectors. In the transitioning countries, this is often due to ad hoc, donor-driven initiatives [29]. Guidance for achieving interoperable health information systems in Botswana should align with the National eHealth Strategy pillar on “Standards and Interoperability” [16]. The strategy seeks to strengthen health information availability and sharing by “Establishing an interoperability architecture” with specific strategic interventions of “Establish[ing] a standards and interoperability framework,” “Design[ing] the interoperability platform,” and “Implement[ing] the interoperability platform” with key stakeholder involvement (subsection 3.5.4, Table number 5 of the National eHealth Strategy [16]).

“Non nocere!” (do no harm) is the indispensable principle of the health care profession, meant to encourage health care practitioners to desist from actions that may result in causing more harm than good [30]. In the age of digital health, the new definition of “do no harm” may include that digital health technologies should “do no harm.” This could be a possible explanation why the HDG principle of “Protect People” may have resonated more with the participants resulting in none of them ranking it as being of lowest priority.

The HDG principles align with consideration for HDG by the WHO Global Strategy on Digital Health [19] and the Africa Health Strategy (2016-2030) by the African Union Commission [20]. Thus, a possible reason why the majority of participants also agreed that the principles in their current form are also adequate in the context of Botswana.

Globally, stakeholders in the health sector are guided and motivated by instruments such as the UHC [9] and the WHO Global Strategy on Digital Health (2020-2025) [19], both of which align with the HDG principles. Consequently, this might have influenced participants’ view of the HDG principles as being of equal importance and priority.

Suggested modifications to the HDG principles by participants were that a clear definition of health data and emphasis on aspects relating to health data accountability and ownership be provided for in the principles (Textbox 1). Health data can be explained as data concerning health [31]. As such health data not only cover specific details of medical conditions, tests, or treatment but also include any related data that reveal anything about an individuals’ past, current, or future health status [32]. This includes both “ill-health” data and “healthy-health” data [31]. With such a broad definition, it is therefore justifiable that the HDG principles should outline what they deem as health data so as to guide any organization willing to implement the principles. Although the HDG principles cite issues relating to health data accountability and ownership, some participants’ sentiments were that such coverage was inadequate and almost suggesting that the HDG principles should be remodeled such that health data accountability and ownership should appear as an additional principle. Other than medical and health records, health data are also increasingly generated through the internet, social media, health apps, and wearable monitors [33]. This health data ecosystem creates a problem that complicates issues related to health data accountability and ownership. Mirchev et al [34] describe this as an under-researched interdisciplinary problem, incorporating legal, ethical, medical, and aspects of ICT. As such further emphasis by the HDG principles on issues relating to health data ownership and accountability may be a worthy consideration.

Themes emerging from Textbox 2 suggest that the successful implementation of the HDG principles will result in a change process within the implementing organization, necessitating organizational readiness and change management strategies [35]. Nilsen et al [36] note that the characteristics of successful changes in a health care organization are having the opportunity to influence the change, being prepared for the change, and valuing the change. This highlights that the implementation of the HDG principles may also require organizational collective motivation [37].

### Future Guidance

Data are an important corporate financial asset [38]. According to Alofaysan et al [39], “organisations should understand that data can lead to better healthcare decisions, which ultimately lead to better business, shifts organisations to a new era of consuming patients’ data rather than only producing it.” It is therefore essential for organizations to implement the HDG principles while being guided by organizational governance structures. The Pan American Health Organization in collaboration with the WHO notes that effective governance will give the individuals in an organization an understanding of the strategic view and the reasoning behind what is being done and why [40], in essence, giving organizational members an understanding of the project aim, direction, and tasks involved in the execution, reducing confusion that might otherwise lead to resistance toward the implementation [40].

Based on these and current study findings, the following guidance for implementing the HDG principles in Botswana is proffered:

- A comprehensive health sector needs assessment is an important first step toward implementation of the HDG principles.
- Considering the sensitive nature of health data, issues of health data security and protection in Botswana should be prioritized and aligned with the DPA (2018) [25].
- Botswana health care sector should engage in relevant and continuous health workforce capacity development to ensure effective and sustainable implementation of the HDG principles.
- The current digital health landscape of Botswana calls for consideration regarding the adoption of eHealth interoperability standards as a cornerstone to the implementation of the HDG principles.

### Limitations

The health sector in Botswana is primarily government-led; therefore, fewer private stakeholders were included in the study compared with public sector stakeholders. Similarly, the understanding of the HDG principles was not the same across public and private sectors; therefore, comparing the two was limited. The views presented on this study might not necessarily be generalizable to the Botswana landscape based on the number of participating organizations. It is also worth noting that some participants might not have been aware or previously exposed to the concept of HDG.

### Conclusions

HDG is an essential part of meeting requirements for UHC. The health sector stakeholders who participated in the study not only acknowledge the relevance and applicability of the HDG principles by Transform Health in the context of Botswana but also suggested some improvements. The existence of other HDG frameworks calls for their critical analysis to assess the most appropriate and applicable framework in the context of Botswana and similar transitioning countries. An organization-centered approach may be most appropriate, as well as strengthening of existing organizations’ HDG practices with the Transform Health principles.

## Abbreviations

DGI: Data Governance Institute
DPA: Data Protection Act
HDG: health data governance
ICT: information and communication technologies
MOH: Ministry of Health
REDCap: Research Electronic Data Capture
UHC: Universal Health Coverage
WHO: World Health Organization

## Data Availability

The data sets used or analyzed during the study are available from the corresponding author on reasonable request.
